# Disruption of ER‐mitochondria tethering and signalling in *C9orf72*‐associated amyotrophic lateral sclerosis and frontotemporal dementia

**DOI:** 10.1111/acel.13549

**Published:** 2022-01-13

**Authors:** Patricia Gomez‐Suaga, Gábor M. Mórotz, Andrea Markovinovic, Sandra M. Martín‐Guerrero, Elisavet Preza, Natalia Arias, Keith Mayl, Afra Aabdien, Vesela Gesheva, Agnes Nishimura, Ambra Annibali, Younbok Lee, Jacqueline C. Mitchell, Selina Wray, Christopher Shaw, Wendy Noble, Christopher C. J. Miller

**Affiliations:** ^1^ Department of Basic and Clinical Neuroscience Institute of Psychiatry, Psychology and Neuroscience King’s College London London UK; ^2^ Department of Neurodegenerative Disease University College London Queen Square Institute of Neurology London UK; ^3^ UK Dementia Research Institute at King's College Institute of Psychiatry, Psychology and Neuroscience King’s College London London UK; ^4^ Present address: Department of Biochemistry, Molecular Biology and Genetics Facultad de Enfermería y Terapia Ocupacional Biomedical Research Networking Center on Neurodegenerative Diseases (CIBERNED) University of Extremadura Cáceres Spain

**Keywords:** amyotrophic lateral sclerosis, *C9orf72*, endoplasmic reticulum, frontotemporal dementia, GSK3β, mitochondria, PTPIP51, VAPB

## Abstract

Hexanucleotide repeat expansions in *C9orf72* are the most common cause of familial amyotrophic lateral sclerosis (ALS) and frontotemporal dementia (FTD). The mechanisms by which the expansions cause disease are not properly understood but a favoured route involves its translation into dipeptide repeat (DPR) polypeptides, some of which are neurotoxic. However, the precise targets for mutant *C9orf72* and DPR toxicity are not fully clear, and damage to several neuronal functions has been described. Many of these functions are regulated by signalling between the endoplasmic reticulum (ER) and mitochondria. ER‐mitochondria signalling requires close physical contacts between the two organelles that are mediated by the VAPB‐PTPIP51 ‘tethering’ proteins. Here, we show that ER‐mitochondria signalling and the VAPB‐PTPIP51 tethers are disrupted in neurons derived from induced pluripotent stem (iPS) cells from patients carrying ALS/FTD pathogenic *C9orf72* expansions and in affected neurons in mutant *C9orf72* transgenic mice. In these mice, disruption of the VAPB‐PTPIP51 tethers occurs prior to disease onset suggesting that it contributes to the pathogenic process. We also show that neurotoxic DPRs disrupt the VAPB‐PTPIP51 interaction and ER‐mitochondria contacts and that this may involve activation of glycogen synthase kinases‐3β (GSK3β), a known negative regulator of VAPB‐PTPIP51 binding. Finally, we show that these DPRs disrupt delivery of Ca^2+^ from ER stores to mitochondria, which is a primary function of the VAPB‐PTPIP51 tethers. This delivery regulates a number of key neuronal functions that are damaged in ALS/FTD including bioenergetics, autophagy and synaptic function. Our findings reveal a new molecular target for mutant *C9orf72*‐mediated toxicity.

## Graphical abstract

Model depicting mechanisms linking ALS/FTD‐mutant *C9orf72* with disruption of the VAPB‐PTPIP51 interaction, synaptic activity and neurodegeneration. *C9orf72*‐derived toxic DPRs activate GSK3β leading to breaking of the VAPB‐PTPIP51 tethers. This perturbs IP3 receptor‐mediated delivery of Ca^2+^ from ER to mitochondria to damage synaptic function and induce neurodegeneration. ALS/FTD linked TDP‐43 and FUS also disrupt the VAPB‐PTPIP51 interaction via activation of GSK3β (*Nature Communications*, 5, 3996; *EMBO Reports*, 17, 1326).

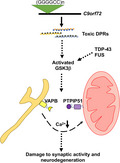

## 1 INTRODUCTION

Amyotrophic lateral sclerosis is the most common form of motor neuron disease and is clinically, genetically and pathologically linked to FTD (also known as frontotemporal lobar degeneration), which is the second most common form of early onset dementia after Alzheimer's disease (Ling et al., 2013). Thus, approximately 15% of FTD patients display clinical ALS features and up to 15% of ALS patients develop symptoms consistent with a clinical definition of FTD (Ling et al., 2013). Likewise, both diseases have a genetic overlap and pathogenic variants in the same genes can cause familial dominantly inherited forms of either FTD or ALS (Ling et al., 2013). Finally, both diseases can display similar pathological phenotypes and notably, the accumulation of abnormal aggregates of TAR DNA‐binding protein 43 (TDP‐43) in affected neurons (Ling et al., 2013; Neumann et al., 2006).

There are no cures nor even effective disease‐modifying treatments for ALS or FTD. Strategies for the development of new therapies include correcting damaged cellular and molecular events but this has been hampered by the broad variety of changes seen in ALS and FTD. Thus, damage to mitochondria, the endoplasmic reticulum (ER) including activation of the unfolded protein response, Ca^2+^ signalling, lipid metabolism, axonal transport, autophagy and inflammatory responses are all the features of ALS/FTD (Lau et al., 2018; Paillusson et al., 2016). Selecting which of these damaged cell functions to prioritise for drug discovery is therefore challenging. Also, this wide variety of damaged cell functions poses questions for underlying disease mechanisms; how do so many disparate cellular functions become collectively perturbed in ALS/FTD?

Recently, damage to signalling between the ER and mitochondria has provided one possible explanation, and this is because ER‐mitochondria signalling regulates many of the damaged functions seen in ALS/FTD (Lau et al., 2018; Paillusson et al., 2016). ER‐mitochondria signalling requires close physical contact between the two organelles such that up to approximately 20% of the mitochondrial surface is closely apposed to ER membranes (Csordas et al., 2006, 2018; Paillusson et al., 2016). The mechanisms by which these contacts form are not properly understood but it is widely accepted that it involves ‘tethering’ proteins that serve to recruit regions of ER to the mitochondrial surface. One of the best characterised tethers involves an interaction between the integral ER protein vesicle‐associated membrane protein‐associated protein B (VAPB) and the outer mitochondrial membrane protein, protein tyrosine phosphatase interacting protein‐51 (PTPIP51) (De Vos et al., 2012; Stoica et al., 2014).

The VAPB‐PTPIP51 tethers are now known to control a number of key neuronal functions including inositol 1,4,5‐trisphosphate (IP3) receptor‐mediated delivery of Ca^2+^ from ER stores to mitochondria; this delivery is a major regulator of mitochondrial ATP production, bioenergetics, autophagy and synaptic activity (De Vos et al., 2012; Gomez‐Suaga et al., 2017, 2019; Paillusson et al., 2017; Stoica et al., 2014, 2016). Synaptic dysfunction is a unifying feature in all the major neurodegenerative disease including ALS/FTD (Spires‐Jones et al., 2017). There is therefore interest in the role of the VAPB‐PTPIP51 tethers not just in normal cellular function, but in neuronal dysfunction, in ALS/FTD and other neurodegenerative diseases (De Vos et al., 2012; Lau et al., 2020; Paillusson et al., 2017; Stoica et al., 2014, 2016).

A number of genes are linked to inherited forms of ALS/FTD but mutations in the *C9orf72* gene represent by far the most common cause of familial ALS/FTD accounting for approximately 11% of ALS and 13% of FTD cases (Dejesus‐Hernandez et al., 2011; Renton et al., 2011). The disease‐causing mutations involve expansion of an intronic GGGGCC hexanucleotide repeat. The mechanisms by which this expansion leads to disease are not properly understood but a favoured route involves the unexpected bidirectional translation of the hexanucleotide repeat by a process termed repeat‐associated non‐ATG translation (Balendra & Isaacs, 2018; Braems et al., 2020; Cook & Petrucelli, 2019). This generates five different dipeptide repeat proteins (DPRs); poly‐GA, poly‐GP, poly‐GR, poly‐PA and poly‐PR (Balendra & Isaacs, 2018; Braems et al., 2020; Cook & Petrucelli, 2019). Three of these are poly‐GA, poly‐GR and poly‐PR that have been shown to be neurotoxic (Kwon et al., 2014; Mizielinska et al., 2014; Wen et al., 2014). However, the targets for this toxicity are not properly understood, and damage to a variety of organelles and cellular functions has been described (Balendra & Isaacs, 2018; Cook & Petrucelli, 2019).

Here, we show that the VAPB‐PTPIP51 ER‐mitochondria tethers are disrupted in neurons derived from iPS cells from familial ALS patients carrying pathogenic *C9orf72* expansions and in transgenic mice expressing ALS/FTD mutant *C9orf72*. We also show that neurotoxic *C9orf72*‐derived DPRs disrupt the VAPB‐PTPIP51 interaction to perturb IP3 receptor‐mediated delivery of Ca^2+^ from ER stores to mitochondria, a key ER‐mitochondria signalling function. Finally, we show that DPR‐mediated damage to the VAPB‐PTPIP51 tethers involves activation of GSK3β. Our findings thus describe a new molecular target for *C9orf72* and DPR‐mediated toxicity.

## 2 RESULTS

### 2.1 The VAPB‐PTPIP51 tethers and IP3 receptor‐VDAC1 interactions are disrupted in iPS cell‐derived cortical neurons from patients carrying pathogenic *C9orf72* expansions

**FIGURE 1:**
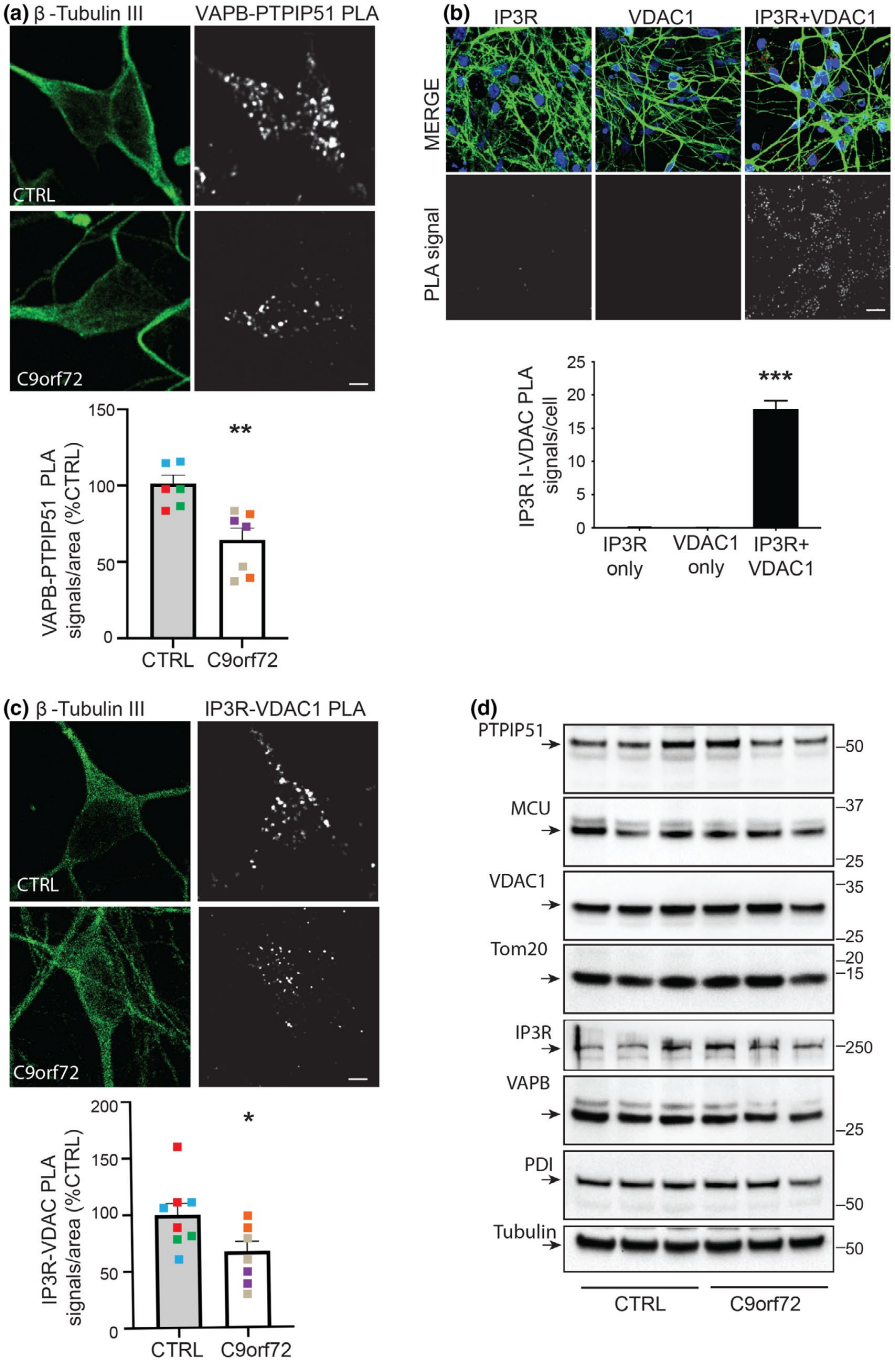
VAPB‐PTPIP51 tethers and IP3 receptor‐VDAC1 interactions are disrupted in iPS cell‐derived neurons from patients carrying pathogenic *C9orf72* expansions but these disruptions do not involve changes in expression of VAPB, PTPIP51, IP3 receptor type 1, VDAC1 or other key ER‐mitochondria signalling proteins. (a, c) Representative projected Z‐stack confocal images of VAPB‐PTPIP51 (a) and IP3 receptor type‐1‐VDAC1 (c) PLAs in control and *C9orf72* patient iPS cell‐derived cortical neurons. Cells were also stained for βIII‐Tubulin to confirm neuronal identity. Scale bars = 5 μm. Bar charts show numbers of PLA signals per cell after correction for cell size and are shown after normalisation to control lines. Quantifications were from three independent healthy control (red, green and blue) and three independent *C9orf72* patient lines (purple, grey and orange), and are from 2 to 3 independent inductions per line. In (a) *n* = 218 control and *n* = 333 *C9orf72* patient neurons. In (c) *n* = 476 control and *n* = 417 *C9orf72* patient neurons. Data were analysed by Mann–Whitney *U* test; *p ≤ 0.05; **p ≤ 0.01. Error bars are standard error of means (SEM). For ease of comprehension, pooled data from all control and *C9orf72* patient cases are shown in the bar charts. However, analyses of the individual patient data also revealed a significant reduction in VAPB‐PTPIP51 PLA signals in each of the *C9orf72* cases compared to controls (***p ≤ 0.001 for all *C9orf72* cases; one‐way analyses of variance (ANOVA), Dunn's multiple comparison test). (b) Control experiments demonstrating the specificity of the IP3 receptor type‐1‐VDAC1 PLAs. Controls included omission of IP3 receptor type 1 (IP3R) or VDAC1 primary antibodies. Samples are counterstained with DAPI to show nuclei and βIII‐Tubulin, showed in MERGE. Graph shows PLA signals per cell. Data were analysed by one‐way ANOVA and Tukey post hoc test. *N* = 37–40 neurons. Error bars are SEM; ***p ≤ 0.001. Scale bar = 30 μm. (d) *C9orf72* iPS cell‐derived neurons do not display changes in expression of VAPB, PTPIP51 or other key ER‐mitochondria Ca^2+^ exchange proteins. Representative immunoblots for VAPB, PTPIP51, VDAC1, IP3R type I, MCU, PDI, TOM20 and βIII‐tubulin as a loading control are shown. Molecular mass markers are indicated in kDa. Signals were normalised to βIII‐tubulin signals, and data were analysed by Mann–Whitney *U* test; no significant differences between control and *C9orf72* lines were detected for any protein

We first studied the effect of mutant *C9orf72* on the VAPB‐PTPIP51 interaction in patient‐derived iPS cell cortical neurons carrying pathogenic GGGGCC expansions. VAPB and PTPIP51 are well‐characterised ER and mitochondrial proteins respectively (Csordas et al., 2018; De Vos et al., 2012; Paillusson et al., 2016). These mutant *C9orf72* iPS cell cortical neurons have been described previously (Simone et al., 2018). For these studies, we analysed three different patients and three different control lines that were derived from healthy individuals (Simone et al., 2018). We used proximity ligation assays (PLAs) to quantify the VAPB‐PTPIP51 interactions; controls to demonstrate the specificity of these VAPB‐PTPIP51 PLAs have been presented in numerous prior studies including on cultured cells, mouse and human tissues (De Vos et al., 2012; Gomez‐Suaga et al., 2019; Lau et al., 2020; Paillusson et al., 2017; Stoica et al., 2016). ER‐mitochondria contacts and the VAPB‐PTPIP51 interaction involve distances of up to approximately 30 nm (Csordas et al., 2006, 2018; Paillusson et al., 2016; Stoica et al., 2014). The distances detected by PLAs are similar to those detected by resonance energy transfer between fluorophores (i.e. a maximum of 30 nm), and so these assays are suitable for quantifying ER‐mitochondria contacts and the VAPB‐PTPIP51 interaction (Paillusson et al., 2016; Soderberg et al., 2006). Indeed, PLAs including ones for VAPB and PTPIP51 have already been used to quantify ER‐mitochondria contacts and signalling (Bernard‐Marissal et al., 2015; De Vos et al., 2012; Gomez‐Suaga et al., 2017, 2019; Hedskog et al., 2013; Lau et al., 2020; Paillusson et al., 2017; Stoica et al., 2016). Moreover, the data generated from these VAPB‐PTPIP51 PLAs have been shown to match data obtained from other assays of ER‐mitochondria contacts including analyses using electron microscopy (Paillusson et al., 2017; Stoica et al., 2014, 2016). Analyses of these iPS cell neuron PLAs revealed that compared to controls, VAPB‐PTPIP51 PLA signal numbers were significantly reduced in *C9orf72* patient neurons (Figure 1a).

As detailed above, a key function of the VAPB‐PTPIP51 tethers is to facilitate delivery of Ca^2+^ from ER stores to mitochondria (De Vos et al., 2012; Gomez‐Suaga et al., 2017; Paillusson et al., 2017; Stoica et al., 2014, 2016). This delivery impacts upon mitochondrial ATP production, bioenergetics, autophagy and synaptic function (Cardenas & Foskett, 2012; Csordas et al., 2018; Gomez‐Suaga et al., 2017, 2019; Paillusson et al., 2016, 2017). Delivery of Ca^2+^ from ER to mitochondria involves its release from ER‐located IP3 receptors and subsequent uptake into mitochondria via the outer mitochondrial membrane located voltage‐dependent anion‐selective channel‐1 (VDAC1) and the inner membrane located mitochondrial calcium uniporter (MCU) (Csordas et al., 2018; Paillusson et al., 2016; Rowland & Voeltz, 2012). IP3 receptor and VDAC1 thus represent a channel for delivery of Ca^2+^ to mitochondria. IP3 receptors and VDAC1 are well‐characterised ER and mitochondrial proteins respectively (Csordas et al., 2018; Lim et al., 2021; Paillusson et al., 2016).

To complement the above VAPB‐PTPIP51 PLA studies, we therefore performed PLAs for IP3 receptors and VDAC1. Similar to the VAPB‐PTPIP51 PLAs described above, control experiments to demonstrate the specificity of the IP3 receptor‐VDAC1 PLAs have been presented in numerous prior studies (Beretta et al., 2020; Bernard‐Marissal et al., 2015; Gomez‐Suaga et al., 2017; Hedskog et al., 2013). There are three subtypes of IP3 receptor (type 1, type 2 and type 3) all of which functions in delivery of Ca^2+^ to mitochondria (Bartok et al., 2019). However, they display differences in expression patterns within the nervous system; IP3 receptor type 1 is highly expressed in neurons in the cortex, hippocampus and cerebellum; IP3 receptor type 2 is mainly expressed in glia and IP3 receptor type 3 is the major isoform in brain stem and spinal cord including motor neurons but is largely absent in cortex and hippocampus (Sharp et al., 1999; Watanabe et al., 2016). We therefore performed PLAs for IP3 receptor type 1 and VDAC1. However, to further check the robustness of these PLAs, we performed control experiments involving omission of one or both primary antibodies; these experiments further demonstrated the specificity of these PLAs in the iPS cell‐derived neurons (Figure 1b). These experiments revealed that compared to controls, IP3 receptor type‐1‐VDAC1 PLA signal numbers per cell were significantly reduced in *C9orf72* patient neurons (Figure 1c).

To determine whether these changes in VAPB‐PTPIP51 and IP3 receptor type‐1‐VDAC1 PLA signals involved alterations in expression of these proteins in the *C9orf72* patient neurons, we performed immunoblots to quantify their expression levels. We also probed the immunoblots for MCU as a further protein involved in Ca^2+^ exchange, for protein disulphide isomerase (PDI) and for translocase of the outer mitochondrial membrane‐20 (TOM20) as general markers for ER and mitochondria proteins, respectively, and for tubulin as a loading control. PDI and TOM20 have been used in previous studies as general ER and mitochondria markers (Gomez‐Suaga et al., 2019; Lau et al., 2020). However, we detected no changes in expression of any of these proteins in the *C9orf72* neurons (Figure 1d). Thus, VAPB‐PTPIP51 and IP3 receptor type‐1‐VDAC1 interactions are both disrupted in mutant *C9orf72* iPS cell‐derived neurons but this disruption does not involve changes in expression of these proteins.

### 2.2 The VAPB‐PTPIP51 interaction is disrupted at an early stage in *C9orf72* transgenic mice carrying a pathogenic repeat expansion

To investigate whether the VAPB‐PTPIP51 interaction is also disrupted by pathogenic *C9orf72* in an in vivo setting, we performed similar VAPB‐PTPIP51 PLAs in the brains of *C9orf72* transgenic mice that carry a bacterial artificial chromosome (BAC) containing a human pathogenic 450 GGGGCC repeat expansion (Jiang et al., 2016). As detailed above, PLAs for the VAPB‐PTPIP51 interaction have been utilised in numerous prior studies including cell, mouse and human tissues, and these studies include control experiments to demonstrate the specificity of the assays (De Vos et al., 2012; Gomez‐Suaga et al., 2019; Lau et al., 2020; Paillusson et al., 2017; Stoica et al., 2016). The *C9orf72* transgenic mice develop age‐dependent GGGGCC RNA foci and perinuclear poly‐GA, poly‐GP and poly‐GR DPR accumulations in multiple regions of the central nervous system (Jiang et al., 2016). However, the mice do not display major motor deficits or spinal cord motor neuron loss. Rather, they develop cognitive and behavioural dysfunctions at 12 months, which are linked to loss of hippocampal neurons (Jiang et al., 2016). We therefore studied the VAPB‐PTPIP51 interaction in the affected CA3 hippocampal region in the *C9orf72* BAC transgenics and non‐transgenic controls at 6 months of age (prior to disease onset) and 12 months of age (early disease).

**FIGURE 2:**
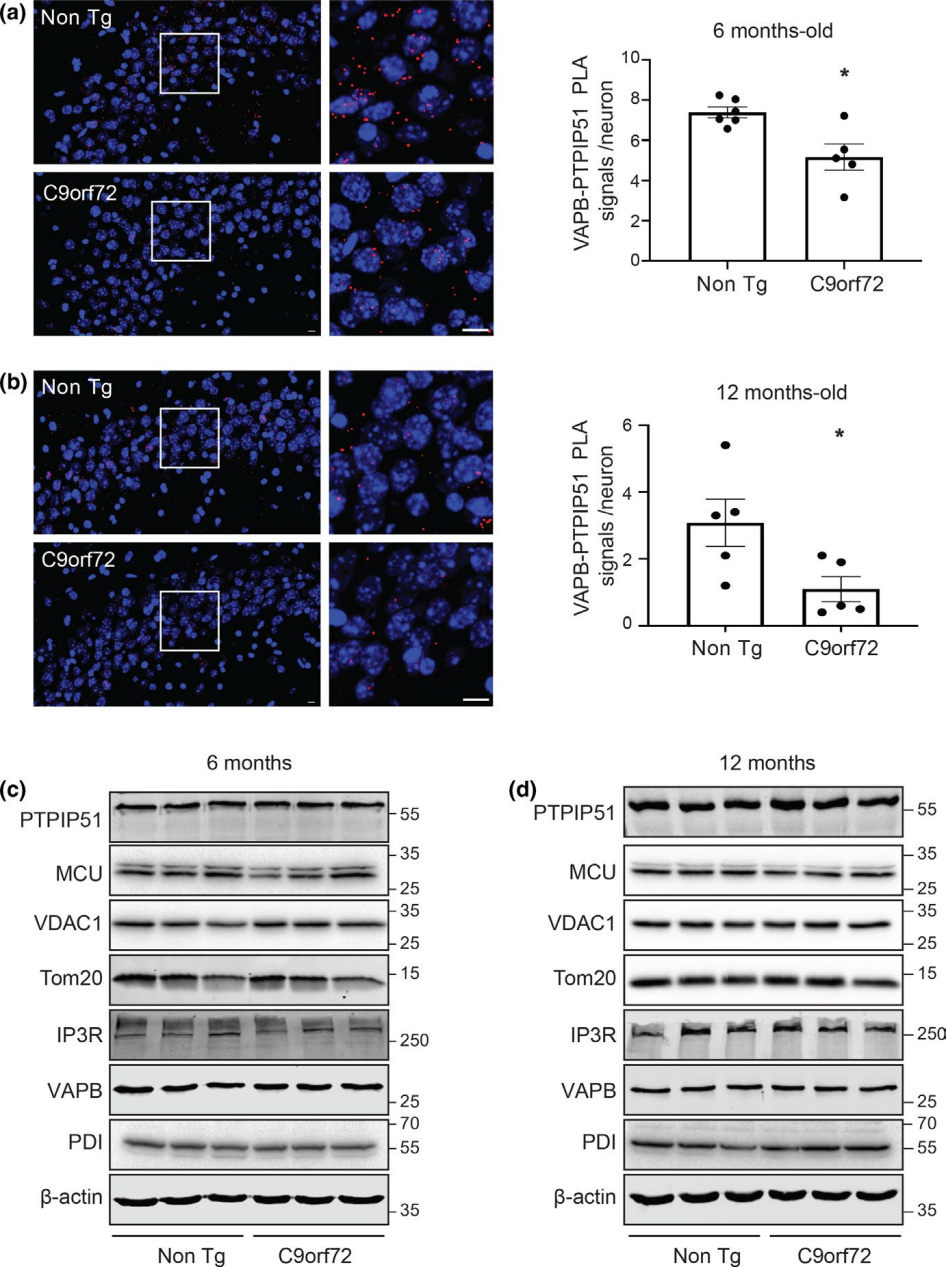
VAPB‐PTPIP51 interaction is disrupted prior to disease onset in affected hippocampal neurons in mutant *C9orf72* transgenic mice but this does not involve changes in expression of brain VAPB, PTPIP51 or other key ER‐mitochondria Ca^2+^ exchange proteins. (a, b) Representative projected Z‐stack confocal images of VAPB‐PTPIP51 PLAs (Red) in *C9orf72* transgenic and non‐transgenic littermate (Non Tg) CA3 hippocampal neurons at 6 and 12 months age as indicated. Samples were also stained with DAPI to show nuclei. Scale bars = 10 μm. Bar charts show numbers of PLA signals per neuron. *N* = 808 cells from 5 *C9orf72* transgenic mice and *N* = 735 cells from 6 non‐transgenic mice in (a) and *N* = 1676 cells from 5 *C9orf72* transgenic mice and *N* = 1370 cells from 5 non‐transgenic mice in (b). Data were analysed by Mann–Whitney *U* test. Error bars are SEM; *p ≤ 0.05. (c, d) *C9orf72* transgenic mice do not display changes in expression of brain VAPB, PTPIP51 or other key ER‐mitochondria Ca^2+^ exchange proteins at either 6 (c) or 12 (d) months of age. Immunoblots for VAPB, PTPIP51, VDAC1, IP3R type 1, MCU, PDI, TOM20 and actin as a loading control are shown. Molecular mass markers are indicated in kDa. Quantifications (not shown) were made from 3 control and 3 *C9orf72* transgenic mice at each age point as indicated. Signals were normalised to actin signals and data were analysed by Mann–Whitney *U* test; no significant differences between control and *C9orf72* lines were detected for any protein

These studies revealed that when compared to non‐transgenic controls, VAPB‐PTPIP51 PLA signals were reduced in mutant *C9orf72* transgenic mice CA3 hippocampal neurons at both 6 and 12 months of age (Figure 2a,b). We also noticed that in control non‐transgenic mice, the numbers of these PLA signals were fewer in 12 month compared to 6‐month‐old mice; the reasons for this age‐dependent reduction are not clear at this stage.

In our study of mutant *C9orf72* patient‐derived iPS cell neurons, we also investigated expression of VAPB, PTPIP51, IP3 receptor type 1, VDAC1, MCU, PDI and TOM20 by immunoblotting in the mice. However, consistent with the findings in the iPS cell neurons, we detected no changes in expression of any of these proteins in the *C9orf72* transgenic mice (Figure 2c,d). Thus, the VAPB‐PTPIP51 interaction is disrupted in affected hippocampal neurons in *C9orf72* transgenic mice carrying a human pathogenic GGGGCC repeat expansion, and this disruption occurs prior to disease onset.

### 2.3 Pathogenic *C9orf72*‐derived DPRs disrupt the VAPB‐PTPIP51 and IP3 receptor‐VDAC interactions, and ER‐mitochondria contacts in cultured rat cortical neurons

**FIGURE 3:**
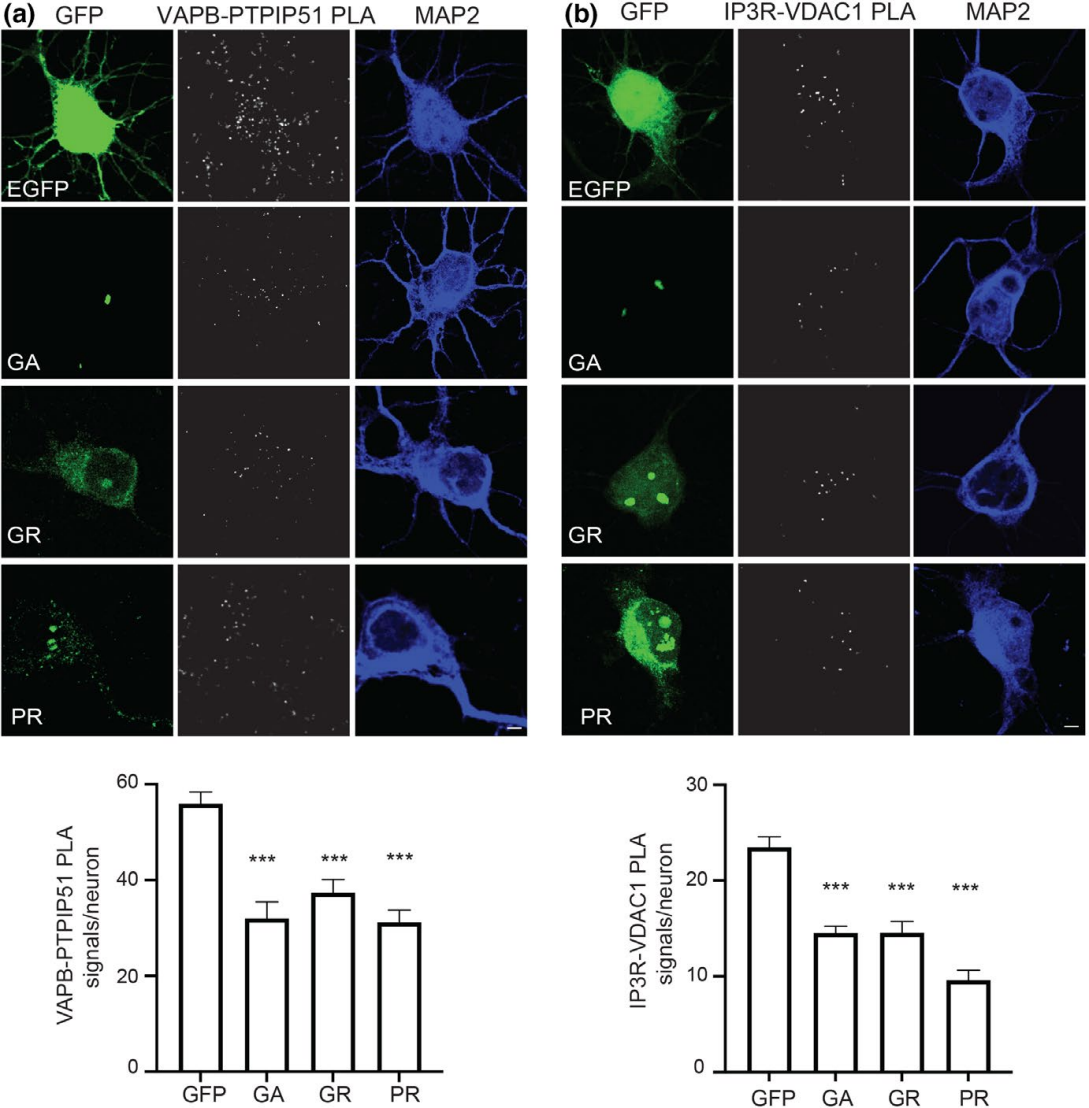
Pathogenic *C9orf72*‐derived DPRs disrupt the VAPB‐PTPIP51 and IP3 receptor‐VDAC1 interactions in rat cortical neurons. Representative projected Z‐stack confocal images of PLAs for (a) VAPB‐PTPIP51 and (b) IP3R‐VDAC1 interactions in rat cortical neurons transfected with either EGFP control vector (GFP), or EGFP fused to poly‐GA, poly‐GR or poly‐PR DPRs. Cells were analysed 16 h post‐transfection. DPRs were detected via their EGFP tags; cells were also stained for MAP2 to confirm neuronal identity (artificially blue). Scale bars = 5 μm. Bar charts shows number of PLA signals per neuron. Data were analysed by one‐way ANOVA with Tukey's post hoc test; (a) *N* = 29–53 neurons from three independent experiments and (b) *N* = 26–71 neurons from three independent experiments. Error bars are SEM; ***p ≤ 0.001

The major pathogenic mechanism for mutant *C9orf72* involves production of DPR proteins derived from the expanded GGGGCC repeats. In particular, three of these DPRs, poly‐GA, poly‐GR and poly‐PR have been shown to be toxic to neurons although the precise mechanism of toxicity is not clear (Balendra & Isaacs, 2018; Kwon et al., 2014; Mizielinska et al., 2014; Wen et al., 2014). We therefore enquired whether these toxic DPRs might disrupt the VAPB‐PTPIP51 and IP3 receptor‐VDAC1 interactions in cultured rat cortical neurons. We chose cortical neurons for investigation since they are affected in *C9orf72*‐linked human FTD. To do so, we transfected cultured rat cortical neurons with enhanced green fluorescent protein (EGFP) control or EGFP‐tagged 125 poly‐GA, poly‐GR and poly‐PR plasmids and again used PLAs to monitor the VAPB‐PTPIP51 and IP3 receptor type‐1‐VDAC1 interactions. These DPRs utilise alternative codon sequences that preclude formation of RNA foci (Lee et al., 2017). Compared to EGFP controls, expression of all three pathogenic DPRs markedly reduced both VAPB‐PTPIP51 and IP3 receptor type‐1‐VDAC1 PLA signals (Figure 3).

**FIGURE 4:**
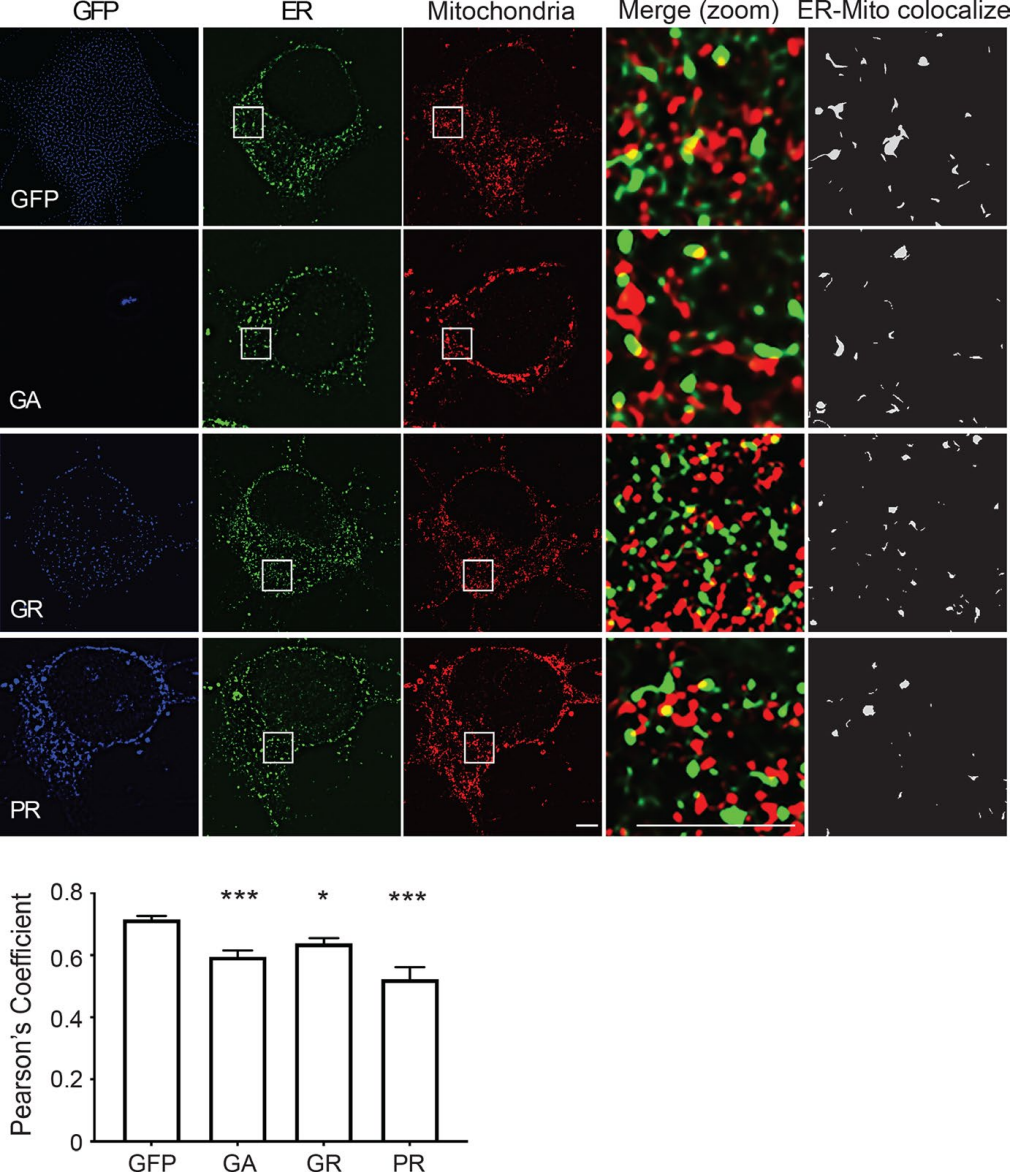
Pathogenic *C9orf72*‐derived DPRs disrupt ER‐mitochondria contacts in rat cortical neurons. Representative single Z‐stack super resolution SIM images of rat cortical neurons transfected with either EGFP control vector (GFP), or EGFP fused to poly‐GA, poly‐GR or poly‐PR DPRs. Cells were analysed 16 h post‐transfection and immunostained for ER (PDI) and mitochondria (TOM20). DPRs were detected via their EGFP tags (shown artificially in blue). Zooms are of boxed regions showing merge and PDI‐TOM20 co‐localised pixels. Scale bars = 5 μm. Bar chart shows ER‐mitochondria (PDI‐TOM20) co‐localisation (Pearson's coefficient). Data were analysed by one‐way ANOVA with Tukey's post hoc test; *N* = 21–34 cells from three independent experiments. Error bars are SEM; *p ≤ 0.05, ***p ≤ 0.001

The VAPB‐PTPIP51 interaction is known to mediate the formation of ER‐mitochondria contacts that are essential for signalling between the two organelles; loss of VAPB or PTPIP51 reduces whereas overexpression of VAPB or PTPIP51 increases ER‐mitochondria contacts (Stoica et al., 2014). We therefore studied the effects of expression of the pathogenic poly‐GA, poly‐GR and poly‐PR DPRs on ER‐mitochondria contacts in the cortical neurons. Transfected neurons were immunostained for PDI and TOM20 as markers for ER and mitochondria, respectively, and ER‐mitochondria co‐localisation then quantified from images acquired using super resolution structured illumination microscopy (SIM). This approach involving PDI‐TOM20 immunostaining and SIM has been used previously to quantify ER‐mitochondria contacts (Gomez‐Suaga et al., 2019; Paillusson et al., 2017; Stoica et al., 2016). In line with the VAPB‐PTPIP51 and IP3 receptor‐VDAC1 PLAs, these analyses revealed that compared to EGFP control, expression of all three pathogenic DPRs significantly reduced PDI‐TOM20 co‐localisation (Figure 4). Thus, expression of mutant *C9orf72*‐derived toxic DPRs reduces the VAPB‐PTPIP51 and IP3 receptor type‐1‐VDAC1 interactions, and ER‐mitochondria contacts.

### 2.4 Pathogenic *C9orf72*‐derived DPRs disrupt ER‐mitochondria Ca^2+^ exchange

A primary function of the VAPB‐PTPIP51 tethers is to facilitate IP3 receptor‐mediated delivery of Ca^2+^ from ER stores to mitochondria (De Vos et al., 2012; Gomez‐Suaga et al., 2017; Paillusson et al., 2017; Stoica et al., 2016). We therefore examined whether the disruption of the VAPB‐PTPIP51 interaction by toxic DPRs, also disrupted this Ca^2+^ exchange. For these experiments, we used DPR‐transfected SH‐SY5Y neuronal cells; SH‐SY5Y cells have been utilised previously to monitor the effects of neurodegenerative disease insults on ER‐mitochondria Ca^2+^ exchange and so represent an established assay system for these experiments (Paillusson et al., 2017).

**FIGURE 5:**
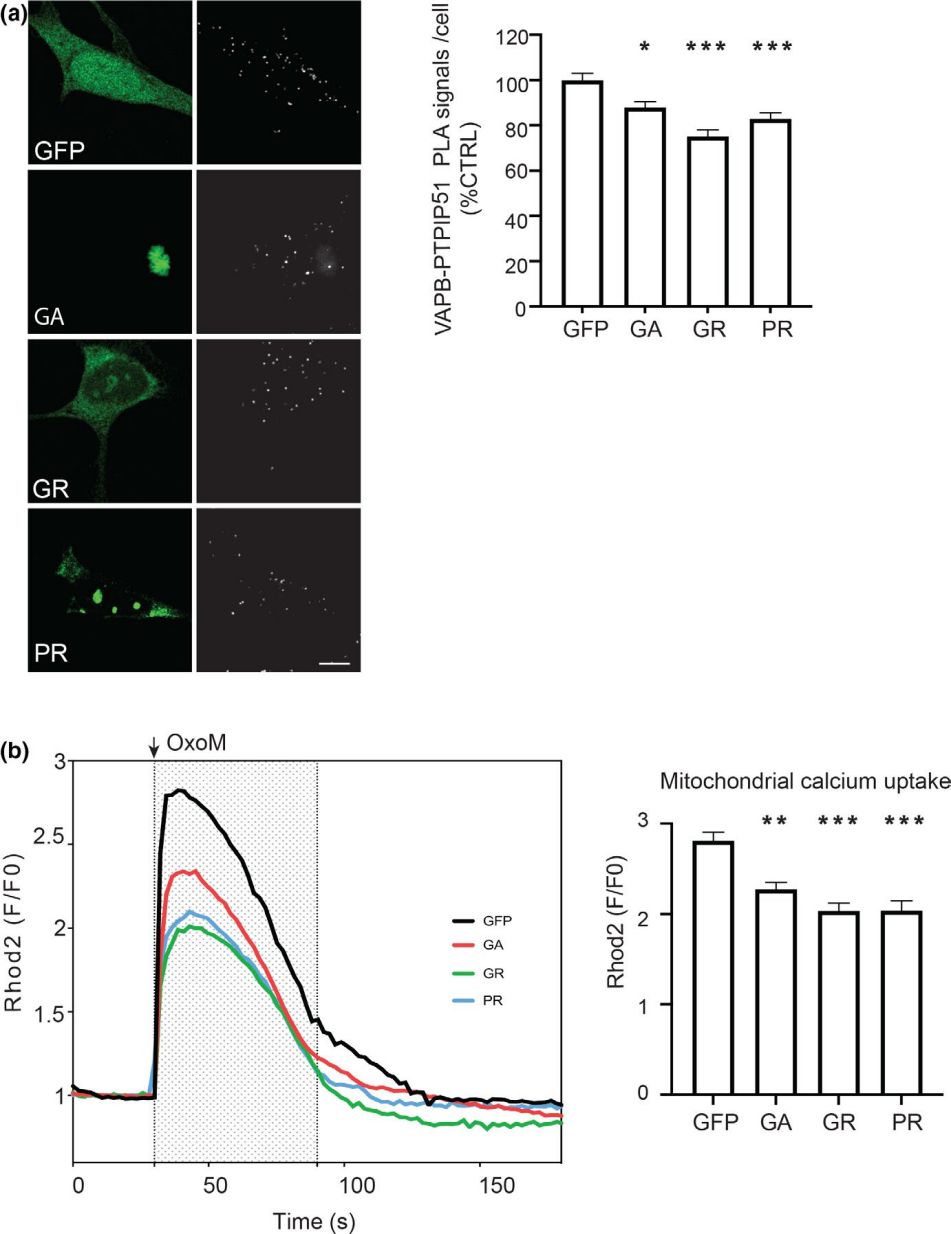
Pathogenic *C9orf72*‐derived DPRs disrupt the VAPB‐PTPIP51 interaction and mitochondrial Ca^2+^ uptake from ER in SH‐SY5Y cells. (a) Pathogenic *C9orf72* DPRs disrupt the VAPB‐PTPIP51 interaction. Representative projected Z‐stack confocal images of VAPB‐PTPIP51 PLAs (white) in SH‐SY5Y cells transfected with either EGFP control vector (GFP), or EGFP fused to poly‐GA, poly‐GR or poly‐PR DPRs. DPRs were detected via their EGFP tags. Bar chart shows quantification of PLAs per cell normalised to EGFP control. Data were analysed by one‐way ANOVA with Tukey's post hoc test. *N* = 108–128 cells from 3 independent experiments. Error bars are SEM; *p ≤ 0.05, ***p < 0.001. Scale bar = 5 μm. (b) Pathogenic *C9orf72* DPRs disrupt mitochondrial Ca^2+^ uptake following IP3 receptor‐mediated release from ER stores. Release of Ca^2+^ was induced by treatment of cells with oxotremorine‐M (OxoM). Mitochondrial Ca^2+^ was detected using Rhod2. Representative Rhod2 fluorescence traces are shown on the left with OxoM treatment depicted by shaded area; normalised peak values are shown in the bar chart on the right. Expression of poly‐GA, ‐GR or ‐PR DPRs all decrease peak mitochondrial Ca^2+^ levels. Data were analysed by one‐way ANOVA and Tukey's post hoc test. *N* = 65–103 cells from three independent experiments. Error bars are SEM; **p ≤ 0.01, **p ≤ 0.001

Firstly, however, we confirmed that expression of pathogenic poly‐GA, poly‐GR and poly‐PR DPRs led to a disruption of the VAPB‐PTPIP51 interaction in SH‐SY5Y cells using PLAs. In line with the results from the rat cortical neurons, compared to EGFP control, expression of EGFP‐poly‐GA, EGFP‐poly‐GR and EGFP‐poly‐PR all induced significant reductions in VAPB‐PTPIP51 PLA signals (Figure 5a).

We next monitored mitochondrial Ca^2+^ levels using the indicator dye Rhod2 following IP3 receptor‐mediated release of Ca^2+^ from ER stores. IP3 receptor‐mediated release was induced by treatment of the cells with the M3 muscarinic acetylcholine receptor agonist oxotremorine‐M. This approach to quantify ER‐mitochondria Ca^2+^ exchange has been utilised previously (De Vos et al., 2012; Gomez‐Suaga et al., 2017; Paillusson et al., 2017; Stoica et al., 2016). In agreement with these earlier studies, oxotremorine‐M induced a time‐dependent increase in mitochondrial Ca^2+^ levels (Figure 5b). However, compared to EGFP‐transfected control cells, the peak values were significantly lower in cells expressing EGFP‐poly‐GA, EGFP‐poly‐GR and EGFP‐poly‐PR (Figure 5b). Thus, disruption of the VAPB‐PTPIP51 tethers by pathogenic *C9orf72*‐derived DPRs is associated with a disruption to ER‐mitochondria Ca^2+^ exchange.

### 2.5 Disruption of the VABP‐PTPIP51 interaction by pathogenic *C9orf72*‐derived DPRs involves activation of GSK3β

**FIGURE 6:**
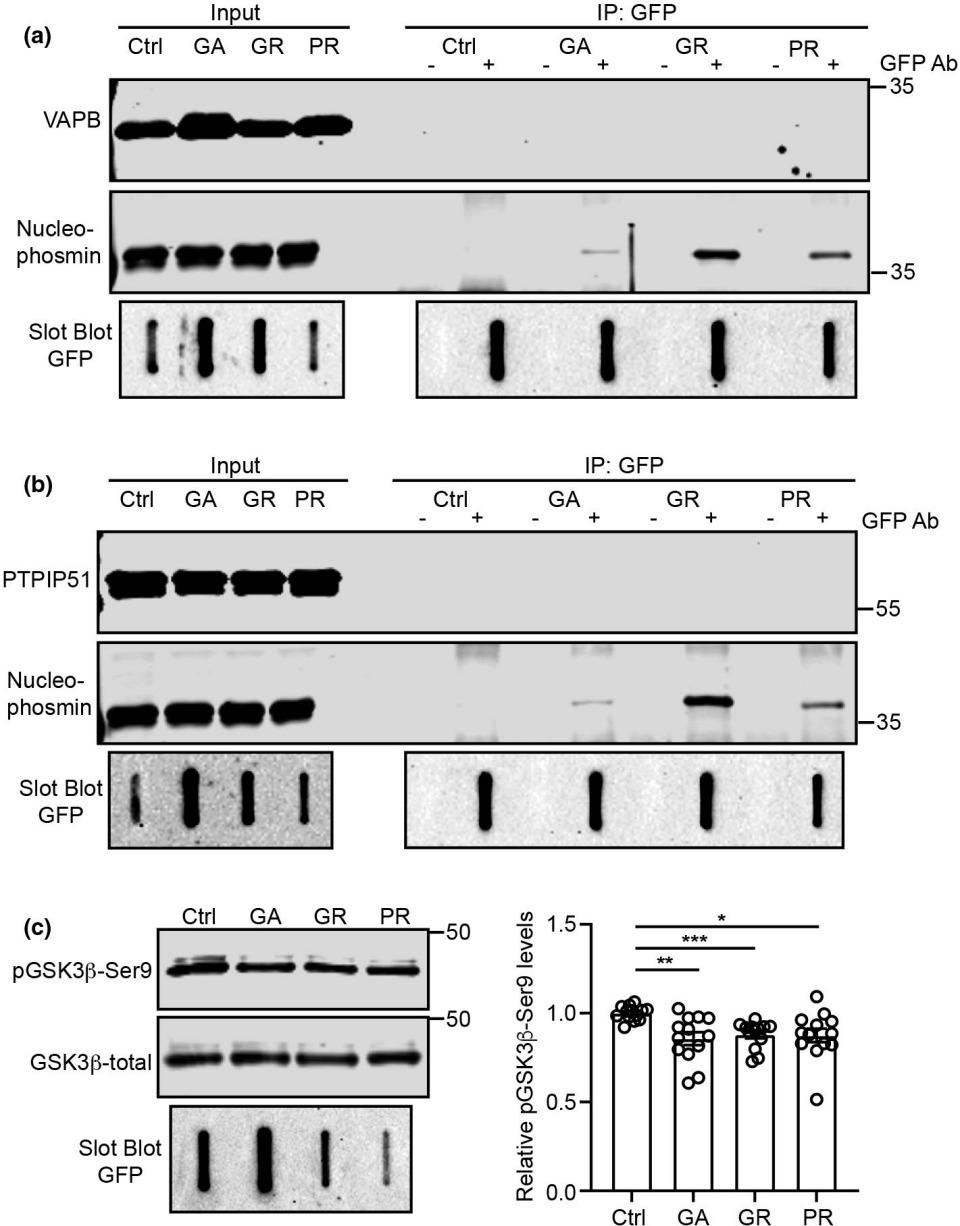
*C9orf72* DPRs do not bind to either VAPB or PTPIP51 but activate GSK3β. (a, b) *C9orf72* DPRs do not bind to either VAPB (a) or PTPIP51 (b). SH‐SY5Y cells were transfected with EGFP control plasmid (Ctrl) or EGFP‐tagged poly‐GA, poly‐GR or poly‐PR DPRs +myc‐VAPB or PTPIP51‐HA as indicated. DPRs were immunoprecipitated using rabbit anti‐GFP antibody and detected using mouse anti‐GFP antibody. VAPB and PTPIP51 were detected using mouse anti‐myc or anti‐HA antibodies to the epitope tags. Positive control endogenous nucleophosmin was detected using mouse anti‐nucleophosmin. DPRs were detected on slot blots; VAPB, PTPIP51 and nucleophosmin were detected after SDS‐PAGE on immunoblots. Both lysate inputs and immunoprecipitates (IP) are shown. (−) and (+) refer to absence or presence of the GFP antibody in the immunoprecipitates; *N* = 3. (c) *C9orf72* DPRs activate GSK3β. SH‐SY5Y cells were transfected with EGFP control plasmid (Ctrl) or EGFP‐tagged poly‐GA, poly‐GR or poly‐PR DPRs and samples probed for total and serine‐9 phosphorylated GSK3β as indicated. Bar chart shows relative levels of serine‐9 phosphorylated inactive GSK3β following normalisation to total GSK3β signals. Data were analysed by Welch ANOVA and Games‐Howell's post hoc test, *N* = 13. Error bars are SEM, *p ≤ 0.05, **p ≤ 0.01, ***p ≤ 0.001

To gain insight into the mechanisms by which the pathogenic *C9orf72*‐derived DPRs ight disrupt the VAPB‐PTPIP51 ER‐mitochondria tethers, we first considered whether the DPRs bound to either VAPB or PTPIP51. There is a precedent for this since Parkinson's disease‐linked α‐synuclein binds to VAPB to disrupt its interaction with PTPIP51 (Paillusson et al., 2017). We therefore transfected cells with EGFP control or EGFP‐poly‐GA, EGPF‐poly‐GR or EGPF‐poly‐PR DPRs, and either VAPB or PTPIP51, immunoprecipitated EGFP and EGFP‐DPRs with EGFP antibody, and probed for the presence of bound VAPB or PTPIP51 in the immunoprecipitates. The C9orf72‐derived DPRs do not resolve properly on SDS‐PAGE, and so, we detected their presence in both the input lysates and immunoprecipitates using slot blots; this approach has been used previously by others (Lee et al., 2017). As a positive control for these experiments, we also probed the samples for endogenous nucleophosmin; nucleophosmin has been shown to bind with poly‐GA, poly‐GR and poly‐PR DPRs in previous studies (Božič et al., 2021; Lee et al., 2016). In line with these previous studies, endogenous nucleophosmin co‐immunoprecipitated with all three DPR proteins. However, we detected no binding of the DPRs with either VAPB or PTPIP51 in these assays (Figure 6a,b).

Although the precise mechanism is not known, GSK3β has been shown to negatively regulate the VAPB‐PTPIP51 interaction and ER‐mitochondria contacts; thus, activation of GSK3β reduces binding of VAPB with PTPIP51, whereas GSK3β inhibitors stimulate the VAPB‐PTPIP51 interaction and ER‐mitochondria contacts (Stoica et al., 2014, 2016). We therefore enquired whether the *C9orf72* DPRs activated GSK3β. A primary mechanism for regulating GSK3β activity involves inhibitory phosphorylation on serine‐9 (Kaidanovich‐Beilin & Woodgett, 2011), and so, we analysed GSK3β serine‐9 phosphorylation by immunoblotting in control EGFP and EGFP‐poly‐GA, EGPF‐poly‐GR and EGPF‐poly‐PR DPR‐transfected SH‐SY5Y cells. These experiments revealed decreased GSK3β serine‐9 phosphorylation in the DPR‐transfected cells (Figure 6c). Thus, mutant *C9orf72*‐derived toxic DPRs activate GSK3β, a known inhibitor of the VAPB‐PTPIP51 interaction and ER‐mitochondria contacts.

## 3 DISCUSSION

Mutations in the *C9orf72* gene involving expansion of an intronic GGGGCC hexanucleotide repeat cause most familial forms of FTD and ALS but the mechanisms by which they induce disease are not properly known. A favoured route involves translation of the repeat into DPR polypeptides, some of which has been shown to be toxic (Balendra & Isaacs, 2018; Braems et al., 2020; Cook & Petrucelli, 2019). However, the expansion can also lead to the formation of repeat containing RNA foci and these may sequester RNA‐binding proteins such as splicing factors and so induce RNA processing defects that could contribute to disease (Balendra & Isaacs, 2018; Cook & Petrucelli, 2019). Finally, the mutations may downregulate expression of C9orf72 protein; C9orf72 is believed to function in autophagy and/or endosomal trafficking, and these processes are known to be damaged in ALS/FTD (Balendra & Isaacs, 2018; Braems et al., 2020; Cook & Petrucelli, 2019). None of these mechanisms are mutually exclusive and all may contribute to disease (Braems et al., 2020).

Irrespective of the precise mechanisms, the targets for pathogenic *C9orf72* expansion‐mediated toxicity are not properly understood. Damage to mitochondria, the ER, autophagy, intracellular transport processes including axonal transport and nucleocytoplasmic trafficking, and Ca^2+^ signaling have all been linked to mutant *C9orf72* (Balendra & Isaacs, 2018; Braems et al., 2020; Cook & Petrucelli, 2019; Dafinca et al., 2016, 2020; Fumagalli et al., 2021). Here, we demonstrate that damage to ER‐mitochondria signalling including disruption of the VAPB‐PTPIP51 tethering proteins is an additional target for this toxicity.

We show that the VAPB‐PTPIP51 tethers are disrupted in human ALS/FTD patient iPS cell‐derived neurons carrying pathogenic *C9orf72* expansions and in transgenic mice expressing a mutant human *C9orf72* gene containing the expanded repeat. In the mice, this damage occurs prior to disease onset. Early pathogenic changes are believed to be the most important; so, this finding supports the notion that disruption of the VAPB‐PTPIP51 tethers contributes in a major way to disease. Whether the changes to the VAPB‐PTPIP51 interaction we detect in the iPS cells and transgenic mice studied here correlate with any changes to mitochondria or ER numbers or morphologies are not clear. No such changes have so far been reported in the *C9orf72* mice (Jiang et al., 2016), and we detected no noticeable overt differences to these organelles in our preliminary investigations. However, future studies involving electron microscope tomography to address these issues would be worthwhile.

We also demonstrate that neurotoxic *C9orf72*‐derived DPR polypeptides disrupt the VAPB‐PTPIP51 interaction, ER‐mitochondria contacts and IP3 receptor‐mediated delivery of Ca^2+^ from ER stores to mitochondria. This delivery is a key ER‐mitochondria signalling function that impacts upon a range of neuronal functions linked to ALS/FTD. These include bioenergetics and mitochondrial ATP production, autophagy, Ca^2+^ homeostasis and synaptic function (De Vos et al., 2012; Gomez‐Suaga et al., 2017, 2019; Hirabayashi et al., 2017; Paillusson et al., 2017; Stoica et al., 2014, 2016).

Interestingly, others have also reported disruption of Ca^2+^ signalling in iPS cell neurons from patients carrying mutant *C9orf72*, and this has been linked to alterations in expression of two mitochondrial Ca^2+^ uptake proteins, mitochondrial Ca^2+^ uptake protein‐1 and ‐2 (MICU1 and MICU2), which are regulatory subunits of the MCU channel (Dafinca et al., 2016, 2020). The VAPB‐PTPIP51 tethers mediate the primary ER‐mitochondria Ca^2+^ exchange from IP3 receptors to the outer mitochondrial membrane located VDAC1 channel. Thus, mutant *C9orf72* may damage a number of key ER‐mitochondria Ca^2+^ exchange proteins.

In order to gain insight into the mechanisms linking neurotoxic *C9orf72*‐derived DPRs with disruption of the VAPB‐PTPIP51 tethers, we enquired whether the DPRs bound to either VAPB or PTPIP51, or whether they activate the kinase GSK3β. Disruption of the VAPB‐PTPIP51 tethers by Parkinson's disease‐linked α‐synuclein involves its direct binding to VAPB, and GSK3β is a known negative regulator of the VAPB‐PTPIP51 interaction (Paillusson et al., 2017; Stoica et al., 2014, 2016). We did not detect any binding of the DPRs with either VAPB or PTPIP51, and this finding is in agreement with previous proteomic studies that analysed the DPR protein interactome and which likewise did not identify VAPB or PTPIP51 as DPR‐binding proteins (Božič et al., 2021; Lee et al., 2016). Rather, we discovered that expression of the DPRs activated GSK3β. This effect was modest (12%–14% decrease in serine‐9 phosphorylation), which may be due to the transient transfection methods used in these experiments. We obtain about a 50% efficiency of DPR transfection, so as approximately half the cells are non‐transfected, the magnitude of the effect of the DPRs on GSK3β activity will naturally be underestimated (non‐transfected cells that do not express the DPRs will clearly show no affect). However, future studies will be required to confirm the effects of the DPRs on GSK3β activity. These should include assays of GSK3β activity in *C9orf72* patient tissues and especially post‐mortem human cases with short post‐mortem times.

A number of other mutant genes linked to familial FTD and/or ALS have been shown to disrupt ER‐mitochondria contacts and/or mitochondrial Ca^2+^ delivery. These include mutant *SIGMAR1* encoding the Sigma‐1 receptor, mutant *SOD1* encoding Cu/Zn superoxide dismutase‐1 (SOD1), mutant *TARDBP* encoding TDP‐43 and mutant *FUS* encoding fused in sarcoma (Bernard‐Marissal et al., 2015; Dafinca et al., 2020; Gregianin et al., 2016; Stoica et al., 2014, 2016; Watanabe et al., 2016). The Sigma‐1 receptor is an ER protein that functions as a chaperone for IP3 receptors to facilitate delivery of Ca^2+^ to mitochondria; the disease‐causing alterations are loss of function mutations (Bernard‐Marissal et al., 2015; Gregianin et al., 2016; Watanabe et al., 2016). Mutant SOD1 damages ER‐mitochondria signalling via disruption of Sigma‐1 receptor function (Watanabe et al., 2016). TDP‐43 accumulations form the hallmark pathology of ALS/FTD but FUS is now also known to be a widespread pathology of ALS (Spires‐Jones et al., 2017). Akin to our results described here on *C9orf72*, TDP‐43 and FUS both disrupt ER‐mitochondria Ca^2+^ delivery via an effect on the VAPB‐PTPIP51 tethers, and this disruption involves activation of GSK3β (Stoica et al., 2014, 2016).

**FIGURE 7:**
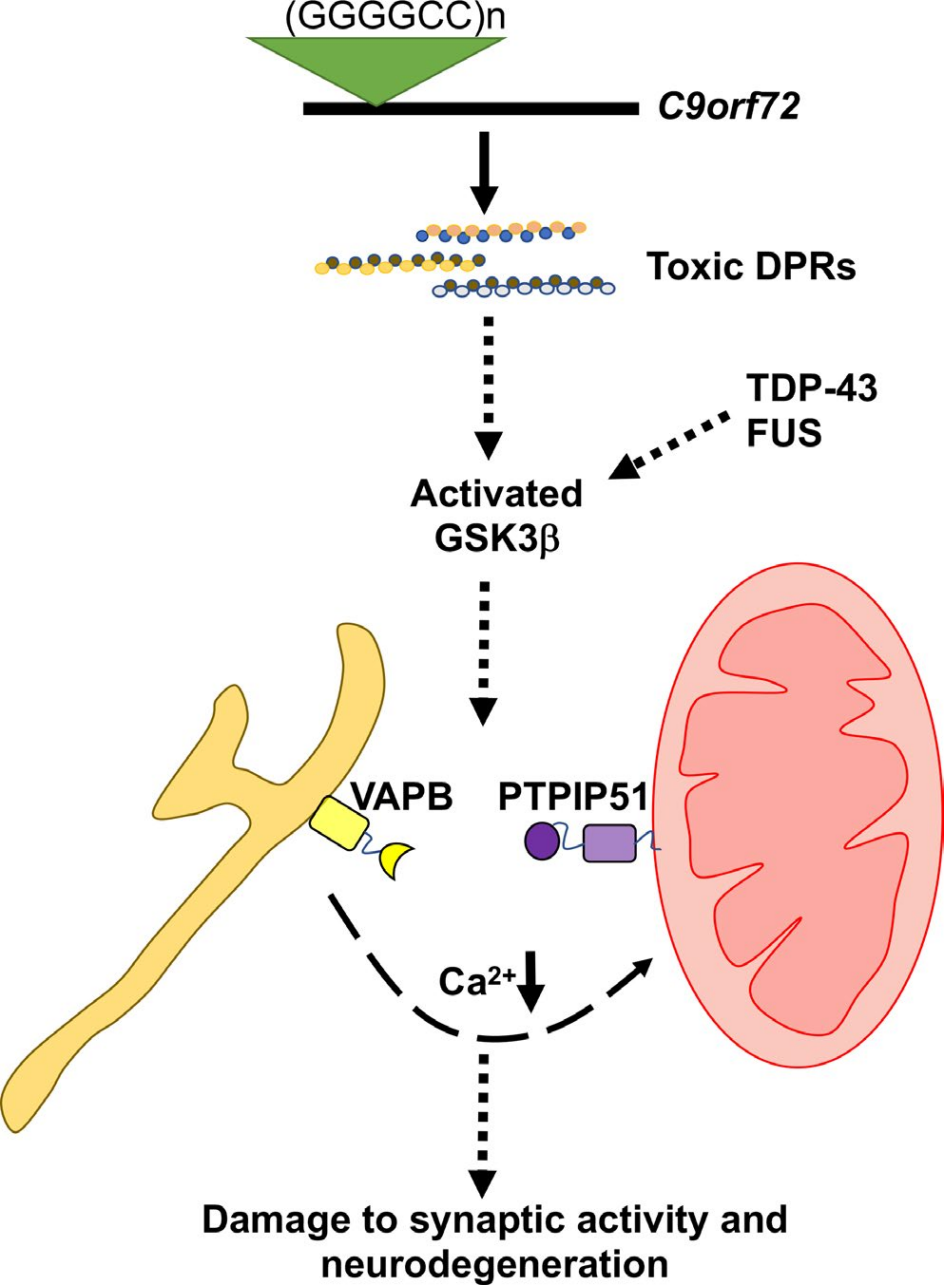
Model depicting mechanisms linking ALS/FTD mutant *C9orf72* with disruption of the VAPB‐PTPIP51 interaction, synaptic activity and neurodegeneration. *C9orf72*‐derived toxic DPRs activate GSK3β leading to breaking of the VAPB‐PTPIP51 tethers. This perturbs IP3 receptor‐mediated delivery of Ca^2+^ from ER to mitochondria to damage synaptic function and induce neurodegeneration. ALS/FTD linked TDP‐43 and FUS also disrupt the VAPB‐PTPIP51 interaction via activation of GSK3β (Stoica et al., 2014, 2016)

In summary, our findings show that ALS/FTD‐associated *C9orf72* damages ER‐mitochondria contacts, IP3 receptor‐mediated delivery of Ca^2+^ to mitochondria and that this damage may involve DPR‐mediated activation of GSK3β and disruption of the VAPB‐PTPIP51 tethering proteins. These findings complement earlier related studies on mutant Sigma‐1 receptor, TDP‐43, FUS and SOD1, which all likewise disrupt ER‐mitochondria contacts and signalling. Thus, five mutant genes linked to familial ALS/FTD have now been shown to target the ER‐mitochondria axis and were studied (*TARDBP*, *FUS* and *C9orf72*); this may involve activation of GSK3β and breaking of the VAPB‐PTPIP51 tethers (Figure 7). Together, they strongly suggest that damage to ER‐mitochondria signalling is a common feature of ALS/FTD. Since ER‐mitochondria signalling regulates many functions perturbed in ALS/FTD, correction of this damage may be broadly therapeutic.

## 4 EXPERIMENTAL PROCEDURES

### 4.1 Plasmids

Expression plasmids for EGFP‐tagged 125 poly‐GA, poly‐GR and poly‐PR DPRs have been described previously; these plasmids utilise alternative codon sequences that preclude formation of RNA foci (Lee et al., 2017). Plasmids were re‐sequenced and sized prior to use to confirm the absence of any rearrangements. Control vector pEGFPC1 was from Clontech. Myc‐tagged VAPB (Myc‐VAPB) and hemagglutinin‐tagged PTPIP51 (PTPIP51‐HA) plasmids in pCIneo have been described previously (Stoica et al., 2014).

### 4.2 Antibodies and other reagents

Rabbit and rat antibodies to VAPB and PTPIP51, respectively, were as previously described (De Vos et al., 2012). Chicken anti‐microtubule‐associated protein‐2 (MAP2) was from Gentex. Rabbit anti‐IP3 receptor type 1 antibodies were from Synaptic Systems and Millipore (for PLAs in mice and neuronal cultures respectively), and from Cell Signaling (for immunoblots). Mouse anti‐MCU (CL3576), mouse anti‐VDAC (20B12AF2) and rabbit anti‐GFP were from Abcam. Mouse anti‐PDI (RL77) was from Thermo Fisher Scientific. Rabbit anti‐TOM20, goat anti‐VDAC1, mouse anti‐GFP (B2) and mouse anti‐total GSK3β (E‐11) were from Santa Cruz Biotechnology. Rabbit anti‐PTPIP51 was from Atlas. Mouse anti‐β‐Tubulin Isotype III and anti β‐actin were from Sigma. Rabbit anti‐serine‐9 phospho‐GSK3β (D85E12), mouse anti‐hemagglutin (HA) epitope tag (6E2) and mouse anti‐myc epitope tag (9B11) were from Cell Signaling. Mouse anti‐nucleophosmin antibody (B23/NPM1) was from ProteinTech. Species‐specific goat and donkey anti‐mouse, anti‐rabbit and anti‐chicken Igs coupled to AlexaFluor‐488, AlexaFluor‐594 or AlexaFluor‐647 were from Invitrogen or Jackson ImmunoResearch. Oxotremorine‐M (OxoM) was from Tocris.

### 4.3 Cell culture and transfection

Cortical neurons were obtained from embryonic day 18 rat embryos. Cortical neurons and SH‐SY5Y cells were cultured and transfected with plasmids using Lipofectamine 2000 as described previously (Morotz et al., 2019; Paillusson et al., 2017). So as to avoid any possible artefacts produced by high levels of expression of EGFP or EGFP‐tagged DPRs in morphology studies, we chose for analyses cells expressing low levels of transfected DPRs as judged by the EGFP signal. This approach has been utilised by us and others in many studies (e.g. Morotz et al., 2019; Vagnoni et al., 2013).

### 4.4 Patient iPS cells and cortical neuron differentiation

Previously described iPS cell lines from two healthy controls and from three *C9orf72* patients were used (Simone et al., 2018). A further control was obtained from the UK stem cell bank. iPS cells were generated, maintained and differentiated into cortical neurons as described (Simone et al., 2018). Karyotyping of iPS cells was performed by Cell Guidance Systems UK. At around days 25–35, neuronal precursors were passaged further with Accutase (Invitrogen) and plated for the final time onto poly‐ornithine and laminin‐coated plates (1 μg/ml) (Sigma), before being used in experiments between days 70 and 75. We detected no significant differences in size between the different genotypes in the iPS cell neurons. However, some neurons in the cultures were larger than others, and so, we corrected for size when presenting the PLA data.

### 4.5 Transgenic mice

*C9orf72* BAC transgenic mice that contain 450 GGGGCC repeat expansions and which were maintained on a C57BL/6 background have been described previously (Jiang et al., 2016). Mice were housed on a 12 h light/dark cycle with ad libitum access to food and water. Mice were analysed at 6 and 12 months of age.

### 4.6 Immunofluorescence staining and proximity ligation assays

Proximity ligation assays were performed essentially as described previously using Duolink In Situ Orange reagents (Sigma‐Aldrich) (De Vos et al., 2012; Gomez‐Suaga et al., 2017; Paillusson et al., 2017). Following PLAs, rat cortical neurons were immunolabeled for MAP2 and iPS cell‐derived cortical neurons labelled for β‐Tubulin Isotype III to confirm neuronal identity. For brain tissues, left brain hemispheres were immersion‐fixed in 4% (w/v) paraformaldehyde in PBS for 24 h at 4 °C, followed by cryoprotection in 30% sucrose in PBS for 24 h at 4 °C. Tissue was embedded and frozen in O.C.T. compound (WVR) and 30 μm cryostat sections prepared. Following PLAs, lipofuscin autofluorescence was quenched by incubating the sections with 0.1% Sudan Black B in 70% ethanol solution. All samples were stained with 300 nM 4′,6‐diamidino‐2‐phenylindole (DAPI) (Thermo Fisher) to stain for nuclei. PLA signals were quantified using the Particle Analysis function of ImageJ.

### 4.7 Microscopy and Ca^2+^ measurements

Confocal microscopy images were acquired using a Leica TCS‐SP5 confocal microscope using a 63x HCX PL APO lambda blue CS 1.4 oil UV objective. Z‐stack images were analysed and processed using Leica Applied Systems (LAS AF6000) image acquisition software. SIM imaging was performed essentially as described previously (Gomez‐Suaga et al., 2019; Paillusson et al., 2017) using Nikon Eclipse Ti‐E Inverted microscope with 100× 1.49 NA CFI objective and equipped with Nikon N‐SIM or Visitech iSIM Super Resolution Systems. Images were captured using an Andor iXon EMCCD camera and reconstructed using Nikon imaging software Elements Advanced Research with N‐SIM module or Nikon deconvolution software for iSIM. ER‐mitochondria contacts were quantified by analyses of PDI/TOM20 co‐localisation with Pearson's coefficient using Nikon Imaging Software Elements Advanced Research.

Ca^2+^ measurements were performed as described previously using 2 μM Rhod2‐AM dye (Invitrogen) (De Vos et al., 2012; Gomez‐Suaga et al., 2017; Stoica et al., 2014, 2016). Rhod2 fluorescence was time‐lapse recorded (1s intervals) at 37 °C with a Nikon Eclipse Ti‐E microscope equipped with a CFI Plan Fluor 40× oil N.A. 1.30 W.D. 0.2 mm spring loaded lens, TiND6 PFS‐S Perfect Focus Unit, Chroma filtersets and Bio‐Logic MSC‐ 200 fast perfusion system. Images were acquired using an Andor Neo sCMOS camera, and data were analysed using Nikon NIS Elements AR software and ImageJ. IP3 receptor‐mediated Ca^2+^ release was triggered by application of 100 μM oxotremorine‐M for 2 min. Mitochondrial Ca^2+^ levels were then calculated as relative Rhod2 fluorescence signals compared to baseline prior to oxotremorine‐M application.

### 4.8 Sodium dodecyl sulphate (SDS)‐polyacrylamide gel electrophoresis (SDS‐PAGE) and immunoblotting

Cultured cells were processed and analysed by SDS‐PAGE and immunoblotting as described previously (Gomez‐Suaga et al., 2017). After probing with primary antibodies, the blots were incubated with horseradish peroxidase conjugated secondary antibodies and developed using chemiluminescence with a Luminata Forte Western HRP substrate system according to the manufacturer's instructions (Millipore). Signals were detected using a BioRad ChemiDoc MP Imaging system. Mouse brains were prepared for SDS‐PAGE as described previously (Stoica et al., 2016), and samples were analysed as above for cultured cells.

### 4.9 Immunoprecipitation assays

Immunoprecipitation assays were performed and analysed by SDS‐PAGE and immunoblotting as described previously (Morotz et al., 2019). Since DPRs do not resolve properly on SDS‐PAGE, DPRs were detected on slot blots essentially as described except using nitrocellulose membranes (Lee et al., 2017). Blots were developed using an enhanced chemiluminescence development reagent (GE Healthcare) and signals detected using a BioRad ChemiDoc MP Imaging system.

### 4.10 Statistical analyses

Statistical analyses were performed using Graphpad Prism 9. Methods of analyses are described in the Figure legends.

## CONFLICT OF INTEREST

The authors declare no conflict of interest.

## AUTHOR CONTRIBUTIONS

PG‐S and CCJM designed the study. PG‐S and CCJM wrote first drafts of the manuscript. PG‐S performed experiments described in Figures 1, 3, 4 and 5 and analysed data. AM, GMM and SMM‐G performed experiments described in Figures 2 and 6 and analysed data. AAnnibali and VG assisted PG‐S with experiments. EP, SW and AN provided and assisted with iPS cells. NA, KM and AAabdien assisted with transgenic mouse samples. JCM, YL, WN and CS assisted in providing reagents and/or expertise. All authors edited the manuscript.

## 5 ETHICAL APPROVAL

All tissue collection and processing were carried out under the regulations and licensing of the Human Tissue Authority, and in accordance with the Human Tissue Act, 2004. Animal experiments were conducted in accordance with the United Kingdom Animals (Scientific Procedures) act 1986.

## Data Availability

Experimental tools and data are available from the corresponding authors.
